# Prevalence and impact of thyroid dysfunction in patients with rheumatoid arthritis

**DOI:** 10.1038/s41598-025-13650-8

**Published:** 2025-10-03

**Authors:** Safa Rahmouni, Malek Dhifallah, Mehdi Mrad, Eya Bouallègue, Khaoula Zouaoui, Asma Krir, Sonia Rekik, Hiba Wahabi, Soumaya Boussaid, Hela Sahli, Afef Bahlous

**Affiliations:** 1 Rheumatology Department, La Rabta Hospital, Tunis, Tunisia; 2 Laboratory of Biochemistry and Hormonology, Institut Pasteur de Tunis, Tunis, Tunisia; 3 Immuno-Rheumatology Research Laboratory LR05SP01, Rheumatology Department, La Rabta Hospital, University of Tunis-El Manar, Tunis, Tunisia

**Keywords:** Anti-thyroid antibody, Autoimmunity, Thyroid dysfunction, Thyroid hormone, Rheumatoid arthritis, Rheumatic diseases, Hormones, Biochemistry, Rheumatology, Endocrinology, Endocrine system and metabolic diseases

## Abstract

Thyroid disorders are common in patients with autoimmune diseases such as rheumatoid arthritis (RA). Both conditions present significant public health concerns due to their impact on quality of life and increased mortality. The aim of this study was to assess the prevalence of thyroid abnormalities in and investigate their influence on rheumatoid arthritis characteristics. This was a case–control study involving adult female patients with RA and age-matched healthy controls. RA disease activity was evaluated using the Disease Activity Score (DAS28), and functional impact was assessed using the Health Assessment Questionnaire (HAQ). Serum thyroid function tests were performed, including thyroid-stimulating hormone (TSH), free thyroxine (FT4), and antithyroid antibodies (TAAs), including anti-thyroglobulin antibodies (TgAb), anti-thyroperoxidase antibodies (TPOAb), and TSH receptor antibodies (TRAb). The study included 58 female RA patients, with a mean age of 52 ± 14.2 years. The median disease duration was 10.41 years. The median DAS28-CRP was 4.03, and the median HAQ was 1.34. The median FT4 and TSH levels were 15.01 [13.48;16.71] and 1.42 [0.91;2.26], respectively. Thyroid dysfunction was identified in 19% of the participants, with hypothyroidism being the most common disorder (17%). Hyperthyroidism was observed in 2% of patients. Antithyroid antibodies were positive in 15.5% (n = 9) of participants, with TPOAb present in 6 patients (10.3%), TgAb in 3 patients (5.2%), and TRAb in 2 patients (3.4%). No statistically significant association was found between thyroid status and RA disease activity, functional impact, or serological status. Despite the lack of correlation, the high prevalence of thyroid dysfunction underscores the importance of regular thyroid screening to optimize management and prognosis in RA patients.

## Introduction

Rheumatoid arthritis (RA) is a chronic autoimmune inflammatory disease of the connective tissue that predominantly affects the synovial membrane, leading to primarily articular manifestations^1^. Although the exact pathophysiological mechanisms remain unclear, RA is considered a multifactorial condition resulting from the interaction between genetic predisposition and environmental triggers, which activate the immune system and promote systemic inflammation^2^. This multifactorial nature of RA may also explain its association with other autoimmune diseases, such as autoimmune thyroiditis^3,4^.

Many studies have investigated the interplay between thyroid function and RA^3,5,6^. A potential association has been suggested between thyroid disorders, particularly hypothyroidism and autoimmune thyroiditis, and the clinical presentation and progression of RA^3^. For instance, hypothyroidism may exacerbate fatigue and musculoskeletal symptoms, mimicking or amplifying the manifestations of RA^7^. Additionally, some studies have reported that the presence of thyroid autoantibodies may correlate with higher disease activity scores^6^, more severe joint damage^8^, or greater functional impairment. However, these findings remain inconsistent, with other studies showing no significant relationship between thyroid status and RA disease outcomes^9,10^.

Although thyroid dysfunction has been widely studied in various populations, data from North African countries, including Tunisia, remain limited. Tunisia is generally considered to have adequate iodine intake due to national salt iodization programs; however, regional variations may still exist^11^. This study aims to address the data gap by providing region-specific insights that could inform local public health approaches. Despite the potential clinical significance of thyroid dysfunction in rheumatoid arthritis (RA), this association has been insufficiently explored in the Tunisian context. We aimed to determine the prevalence of thyroid abnormalities among RA patients and to evaluate their impact on clinical presentation, disease activity, and functional status.

## Methods

### Study design

This case–control study was conducted between December 2021 and November 2022 at the Rheumatology Department of La Rabta hospital, a tertiary care center in Tunis, Tunisia. The study included adult female patients diagnosed with rheumatoid arthritis (RA) according to the 2010 American College of Rheumatology (ACR)/European League Against Rheumatism (EULAR) classification criteria^12^, and a group of healthy controls. Patients were consecutively recruited during routine outpatient visits or hospitalizations. The source population consisted of RA patients regularly followed in our department, representing a typical North African cohort with established RA. The control group consisted of adult female volunteers recruited from hospital staff, non-blood-related companions of patients, and individuals attending routine health screenings in the community. Controls were matched to RA patients by age (± 2 years) to minimize age-related bias. The exclusive inclusion of females aimed to reduce sex-related confounding. Matching was limited to age and did not extend to other variables such as BMI or comorbidities.

Participants with potential confounding factors that could influence thyroid function were excluded. These included individuals with a history of thyroidectomy or cervical irradiation, those receiving medications affecting thyroid function (such as amiodarone, lithium, or corticosteroids above physiological doses), as well as patients with active infiltrative diseases (e.g., tuberculosis, sarcoidosis), pituitary adenomas, pregnancy or breastfeeding, acute or chronic renal failure, chronic liver disease, known malignancies, or ongoing severe infections. All included participants were screened for thyroid function for the first time during this study.

### Data collection

We systematically collected a comprehensive set of data, including demographic information (age and comorbidities), as well as detailed RA characteristics such as the age of onset, disease duration, and the number of swollen and tender joints. We also assessed morning stiffness and nocturnal awakenings to capture the functional burden of the disease. Radiographs of the hands and forefeet were obtained to evaluate the extent of structural damage caused by RA. Disease activity was measured using the Disease Activity Score (DAS28), incorporating C-reactive protein (CRP) as a biomarker for inflammation.

The activity score of patients is graded as follows: Remission (DAS 28 -CRP score of ≤ 2.6), low activity (DAS 28 -CRP > 2.6 and ≤ 3.2), moderate activity (DAS 28 -CRP > 3.2 and ≤ 5.1), and high activity (DAS 28 -CRP > 5.1)^13^.

To assess the functional impact of the disease, we used the Health Assessment Questionnaire (HAQ).

In addition, we documented functional signs related to thyroid dysfunction, which included symptoms indicative of hypothyroidism such as physical fatigue, hair loss, mental fatigue, dry skin, constipation, and weight gain, along with signs of hyperthyroidism (such as weight loss, nervousness, and anorexia).

Immunological assessments were carried out to measure the presence of rheumatoid factor (RF) and anti-citrullinated protein antibodies (ACPA). Furthermore, thyroid function was carefully evaluated through the measurement of thyroid-stimulating hormone (TSH), free thyroxine (FT4), and thyroid autoantibodies (TAAs), including anti-thyroglobulin antibodies (TgAb), anti-thyroid peroxidase antibodies (TPOAb), and TSH receptor antibodies (TRAb).

Thyroid dysfunction was defined as any abnormality in serum TSH levels, with or without variations in FT4 levels. Thyroid autoimmunity was considered present if detectable levels of TPOAb, TgAb, and/or TRAb were identified, with or without accompanying thyroid dysfunction.

### Statistical study

Descriptive statistics were computed for demographic, clinical, biological, radiographic, and therapeutic characteristics. Continuous variables were expressed as mean ± standard deviation (SD) or median (interquartile range, IQR) based on data distribution. Categorical variables were expressed as frequencies and percentages.

The Kolmogorov–Smirnov test was used to assess the normality of the data distribution. For normally distributed data, parametric tests were used, including the Student’s t-test for comparing means between two groups. For non-normally distributed data, non-parametric tests, such as the Mann–Whitney U test, were applied.

Associations between categorical variables were evaluated using the Chi-square test or Fisher’s exact test when the expected cell counts were less than 5. Correlation analyses were performed using Pearson’s or Spearman’s correlation coefficients depending on the normality of the data.

The significance level was set at *p* < 0.05 for all statistical tests.

## Results

We collected data from 58 female patients with RA. As shown in Table 1**.** The mean age was 52.38 ± 14.21 years. The median disease duration was 10.41 years, with more than half (52%) having a disease course of over 10 years. Immunologically, RF was positive in 87% of patients, and ACPA were positive in 73%. Erosive disease was observed in 66% of the patients. The median DAS28-CRP was 4.03, with the following distribution: Remission (8%), low activity (20%), moderate activity (47%), and high activity (25%).

**Table 1:** Demographic and clinical characteristics of RA patients.

Age (years) (mean ± SD)	52.38 ± 14.21
Comorbidities (n (%))	
Diabetes	8 (13.8)
Hypertension	18 (31)
Dyslipidemia	37 (63.8)
Overweight or obesity	35 (60.5)
Characteristics of RA	
Age of onset (years)	41[30.14;51.00]ª
Duration of disease (years)	10.41[3.16;16.11]ª
Duration of disease > 10 years (n (%))	30(52)
DAS28-CRP	4.03[2.95;5.28]ª
Presence of tender joints (n (%))	50 (86)
NTJ	6 [2;10]ª
Presence of swollen joints (n (%))	49 (85)
NSJ	4 [2;7]ª
VAS	5 [3;7]ª
Presence of morning stiffness (n (%))	36(62)
Duration of morning stiffness (minutes)	60[30;120]ª
Presence of nocturnal awakening (n (%))	35(60)
Number of nocturnal awakenings	2 [2;3]ª
HAQ	1.34[0.97;1.50]ª
Functional disability (n (%))	34(92)
Structural damage (n (%))	39(66)
CRP (mg/L)	8.38 [2.23;21.52]ª
Positive RF (n (%))	46(87)
Positive ACPA (n (%))	36(73)

SD, Standard deviation; n, Number; RA, Rheumatoid arthritis; DAS28, Disease activity score; NTJ, Number of tender joints; NSJ, Number of swollen joints; VAS, Visual analog scale; HAQ, Health Assessment Questionnaire; CRP, C-reactive protein; RF, Rheumatoid factor; ACPA, Anti-citrullinated protein antibodies. ª: Median [interquartile range].

The control group included 58 participants, with a mean age of 52.13 ± 14.77 years. There was no statistically significant difference in age between the two groups (*p* = 0.929).

### Assessment of thyroid function tests

Regarding thyroid biological and immunological status, it showed that thyroid dysfunction was observed in 19% of RA patients (Table 2). The majority had hypothyroidism (17%), with 9% having subclinical hypothyroidism and 2% experiencing hyperthyroidism. Autoimmune thyroid disease was present in 15.5% of patients. Regarding thyroid autoantibodies, 10.3% of patients tested positive for TPOAb, 5.2% for TgAb, and 3.4% for TRAb. Compared to the control group, thyroid dysfunction, particularly hypothyroidism and higher levels of thyroid autoantibodies (TPOAb and TgAb), were significantly more frequent in the RA group.

**Table 2:** Biological and immunological thyroid status.

Biological data:	RA group	Control group	*p*-value	Reference values
FT4 (pmol/L)	15.01 [13.48;16.71]	15.4 [13.87;16.66]	0.362	12–22
TSH (mUI/L)	1.42 [0.91;2.26]	1,79 [1.25;2.26]	0.152	0.27–4.2
TPOAb (UI/mL)	12.80 [8.98–16.43]	6.81 [5.19–9.17]	< 0.01	0–34
TgAb (UI/mL)	16.49 [13.32–21.31]	10.36 [10–12.53]	< 0.01	0–115
TRAb (UI/L)	0.9 [0.8–2.7]	0.83 [0.8–2.01]	0.02	< 1.75
Positive TPOAb (n (%))	6 (10.3)	5 (9)	0.751	
Positive TgAb (n (%))	3 (5.2)	3 (5)	1.000	
Positive TRAb (n (%))	2 (3.4)	1 (2)	0.500	
Thyroid status				
Thyroid dysfunction (n (%))	11 (19)	3 (5)	< 0.01	
Hypothyroidism (n (%)) *	10 (17)	3 (5)	0.013	
Hyperthyroidism (n (%))	1 (2)	0 (0)	1.000	
Autoimmune thyroid disease (n (%))	9 (15.5)	**7 (12)**	**0.590**	

FT4, Free thyroxine; TSH, Thyroid-stimulating hormone; TPOAb, Anti-thyroid peroxidase antibodies; TgAb, Anti-thyroglobulin antibodies; TRAb, Anti-TSH receptor antibodies; n, Number.*Hypothyroidism includes overt hypothyroidism and subclinical hypothyroidism.

Functional symptoms related to thyroid dysfunction in RA patients are demonstrated in Table 3.

**Table 3:** Functional symptoms of thyroid dysfunction.

Functional signs	
Signs of hypothyroidism (n (%))	
Physical fatigue	38 (65.5)
Hair loss	20 (34.5)
Mental fatigue	19 (32.8)
Dry skin	15 (25.9)
Constipation	11 (19)
Weight gain	9 (15.5)
Eyebrow and axillary hair loss	6 (10.3)
Cramps	4 (6.9)
Sensitivity to cold	4 (6.9)
Signs of hyperthyroidism (n (%))	
Physical or mental fatigue	35 (60.3)
Weight loss	16 (27.6)
Nervousness	15 (25.9)
Anorexia	15 (25.9)
Palpitations	13 (22.4)
Diarrhea	6 (10.3)
Hand tremors	3 (5.2)
Hand sweating	1 (1.7)

n: Number The most common symptoms of hypothyroidism were physical fatigue (65.5%), hair loss (34.5%), and mental fatigue (32.8%). Other notable symptoms included dry skin (25.9%), constipation (19%), and weight gain (15.5%). Additionally, 6.9% of patients reported eyebrow and axillary hair loss, cramps, and sensitivity to cold. On the other hand, symptoms of hyperthyroidism were less prevalent, with physical or mental fatigue reported in 60.3% of cases, followed by weight loss (27.6%) and nervousness (25.9%). Other signs of hyperthyroidism included anorexia, palpitations, and diarrhea, with a smaller percentage of patients experiencing hand tremors and hand sweating.

### Comparison of clinical and demographic characteristics of rheumatoid arthritis patients according to thyroid status

As exhibited in Table 4, the comparison of disease characteristics between RA patients with hypothyroidism and euthyroidism showed no significant differences in various clinical and demographic parameters. The mean age was similar between the two groups (54.3 ± 13.7 years versus 51.6 ± 14.4 years, *p* = 0.585), and there were no significant differences in the prevalence of hypertension, diabetes, dyslipidemia, or overweight/obesity. Disease duration was comparable between hypothyroid and euthyroid patients (13.13 versus 9.19 years, p = 0.428). Similarly, disease activity (DAS28-CRP: 4.49 [3.25–5.28] vesus 3.92 [2.95–5.26], *p* = 0.629), swollen joint count (SJC: 5.6 ± 5.6 versus 5.2 ± 5.2, *p* = 0.814), tender joint count (TJC: 7 ± 5.63 versus 6.9 ± 6.6, *p* = 0.956), C-reactive protein levels (CRP: 11.8 ± 7.8 versus 20.8 ± 34.2, *p* = 0.414), and pain intensity (VAS: 4.6 ± 2.2 versus 5.3 ± 2.4, *p* = 0.428) were also similar between groups. Functional impact, assessed by HAQ, was identical in both groups (1.5, *p* = 0.690). The prevalence of positive ACPA, ANA, and RF was not significantly different between the groups.

**Table 4:** Comparison of clinical and demographic characteristics of rheumatoid arthritis patients according to thyroid status (hypothyroidism vs. Euthyroidism).

		Hypothyroidism (n = 10)	Euthyroidism (n = 47)	p
Age (years) (mean ± SD)		54.3 ± 13.7	51.6 ± 14.4	0.585
Hypertension	Yes	3 (30%)	15 (32%)	1.000
	No	7 (70%)	32 (68%)	
Diabetes	Yes	0 (0%)	9 (19%)	1.000
	No	10 (100%)	38 (81%)	
Dyslipidemia	Yes	6 (60%)	30 (64%)	1.000
	No	4 (40%)	17 (36%)	
Overweight or obesity	Yes	7 (70%)	28 (60%)	1.000
	No	3 (30%)	19 (40%)	
Age of onset (years)		44[37; 53]	40[29.67;49.00]	0.326
Duration of disease (years)		13,13 [1,13; 14,32]	9,19[3,16; 21,17]	0.428
DAS28 crp		4.49 [3.25–5.28]	3.92 [2.95–5.26]	0.629
NSJ		5.6 ± 5.6	5.2 ± 5.2	0.814
NTJ		7 ± 5.63	6.9 ± 6.6	0.956
CRP		11.8 ± 7.8	20.8 ± 34.2	0.414
VAS		4.6 ± 2.2	5.3 ± 2.4	0.428
HAQ		1.5 [1.38–1.75]	1.5 [1.2–1.75]	0.690
ACPA	Positive	8 (80%)	27 (57%)	0.287
	Negative	2 (20%)	20 (43%)	
ANA	Positive	2 (20%)	9 (19%)	1.000
	Negative	8 (80%)	38 (81%)	
RF	Positive	8 (80%)	37 (79%)	1.000
	Negative	2 (20%)	10 (21%)	
Erosions	Present	7 (70%)	16 (33.3%)	1.000
	Absent	3 (30%)	32 (66.6%)	

N, Number; p, Significance; SD, Standard deviation; DAS28, Disease activity score, NSJ, Number of swollen joints; NTJ, Number of tender joints; CRP, C-Reactive protein; VAS, Visual Analogue Scale; HAQ, Health Assessment Questionnaire; ACPA, Anti-cyclic citrullinated peptide antibodies; ANA, Antinuclear antibodies; RF, Rheumatoid factor.

### Correlation between thyroid hormones, thyroid autoantibodies, and clinical-biological parameters of rheumatoid arthritis

As shown in Table 5, the analysis of correlations between thyroid hormones (FT4, TSH), thyroid autoantibodies (TPOAb, TgAb, TRAb), and clinical-biological parameters of rheumatoid arthritis (RA) revealed no statistically significant associations. Disease duration, disease activity (DAS28), tender joint count, swollen joint count, patient global assessment (VAS), inflammation markers (CRP), as well as structural (NAD, NAT) and global (EGP) assessment parameters, all showed weak and non-significant correlations with thyroid markers. Notably, TRAb levels had a marginally stronger, though still non-significant, correlation with HAQ (*p* = 0.072).

**Table 5:** Correlations between thyroid hormones, autoimmune thyroid antibodies, and clinical-biological parameters of rheumatoid arthritis.

		FT4	TSH	TPOAb	TgAb	TRAb
Duration of disease (years)	*r*	− 0.006	− 0.226	− 0.196	0.153	0.026
	*p*	0.966	0.091	0.144	0.256	0.847
DAS28	*r*	− 0.061	− 0.013	0.064	− 0.094	0.175
	*p*	0.647	0.922	0.633	0.484	0.189
NTJ	*r*	− 0.055	− 0.154	− 0.024	− 0.079	0.171
	*p*	0.682	0.248	0.855	0.557	0.200
NSJ	*r*	− 0.034	− 0.34	0.022	− 0.042	0.194
	*p*	0.802	0.801	0.870	0.755	0.144
VAS	*r*	− 0.205	0.025	0.027	− 0.059	0.055
	*p*	0.123	0.852	0.840	0.662	0.682
HAQ	*r*	0.010	− 0.062	− 0.008	− 0.026	0.300
	*p*	0.955	0.715	0.963	0.877	0.072
CRP	*r*	− 0.056	0.026	− 0.016	− 0.042	0.044
	*p*	0.678	0.846	0.904	0.754	0.745

FT4, Free thyroxine; TSH, Thyroid-stimulating hormone; TPOAb, Anti-thyroid peroxidase antibodies; TgAb, Anti-thyroglobulin antibodies; TRAb, Anti-TSH receptor antibodies; r, Pearson’s correlation coefficient; p, *p*-value; DAS28, Disease Activity Score; NTJ, Number of tender joints; NSJ, Number of swollen joints; VAS, Visual Analogue Scale; HAQ, Health Assessment Questionnaire; CRP, C-reactive protein.

Similarly in Table 6, across all groups (positive vs. negative for each type of thyroid autoantibody), there were no statistically significant differences observed in disease duration, DAS28, HAQ, and CRP.

**Table 6:** Comparison of rheumatoid arthritis characteristics according to thyroid autoantibody status (AAT, TPOAb, TgAb, and TRAb).

	Positive AAT (n = 9)	Negative AAT (n = 49)	p
Duration of disease (years)	11,46 ± 7,74	11.59 ± 8.96	0.878
DAS28	3.67 ± 1.89	4.28 ± 1.48	0.334
HAQ	1.42 ± 0.57	1.55 ± 0.66	0.321
CRP	13.72 ± 12.33	20.24 ± 33.7	0.813
Erosions (n (%))	7 (77.8)	32 (65.3)	0.703

	Positive TPOAb (n = 6)	Negative TPOAb (n = 52)	p
Duration of disease (years)	7.89 ± 5.21	12 ± 8.97	0.352
DAS28	3.54 ± 2.2	4.26 ± 1.46	0.402
HAQ	1.45 ± 0.64	1.54 ± 0.65	0.449
CRP	10.9 ± 11.65	20.19 ± 32.82	0.645
Erosions (n (%))	4 (66.7)	35 (67.3)	1.000

	Positive TgAb (n = 3)	Negative TgAb (n = 55)	p
Duration of disease (years)	15.59 ± 11.56	11.34 ± 8.62	0.399
DAS28	3.67 ± 1.66	4.22 ± 1.55	0.617
HAQ	1.4 ± 0.14	1.53 ± 0.66	0.631
CRP	15.87 ± 5.86	19.4 ± 32.2	0.307
Erosions (n (%))	2 (66.7)	37 (66.3)	1.000

	Positive TRAb (n = 2)	Negative TRAb (n = 56)	p
Duration of disease (years)	14.25 ± 0.13	11.47 ± 8.86	0.476
DAS28	4 ± 2.05	4.2 ± 1.55	0.818
HAQ	–	–	–
CRP	20.92 ± 19.53	19.17 ± 31.82	0.460
Erosions (n (%))	2 (100)	37 (66.1)	1.000

AAT, Antithyroid antibodies; n, Number; p, Significance; DAS28, Disease Activity Score; HAQ, Health Assessment Questionnaire; CRP, C-reactive protein; TPOAb, Anti-thyroid peroxidase antibodies; TgAb, Anti-thyroglobulin antibodies; TRAb, Anti-TSH receptor antibodies.

## Discussion

In our study, thyroid dysfunction was found in 19% of patients with rheumatoid arthritis, with hypothyroidism (clinical and subclinical) being the most common disorder, affecting 17% of patients. Hyperthyroidism was present in 2% of patients. Positive thyroid autoantibodies were detected in 16% of patients, with positivity rates of 10.3% for TPOAb, 5.2% for TgAb, and 3.4% for TRAb. Despite the presence of these autoantibodies, no significant differences were observed between patients with euthyroid and hypothyroid states in terms of disease duration, disease activity (DAS28), functional impact (HAQ), tender joint count, swollen joint count, patient global assessment (VAS) or inflammation markers (CRP). Additionally, no significant correlations were found between thyroid hormone levels, thyroid autoantibodies, and the clinical or biological parameters of RA. Notably, TRAb levels had a marginally stronger, though still non-significant, correlation with HAQ (*p* = 0.072).

The relation between RA and thyroid function has long been studied^14,15^. In fact, both disorders share common genetic and environmental factors involved in their pathogenesis, leading to a breakdown in self-tolerance and the development of autoimmune diseases. Several studies have suggested the involvement of shared genes in the susceptibility to developing both RA and autoimmune thyroid disorders. For instance, HLA-DRB1 (Human Leukocyte Antigen DR) is considered a major susceptibility locus for RA^14,16^, while arginine at position 74 of the HLA-DRB1 chain (DRb-Arg74) has been implicated in the development of Graves’ disease^17^. The role of thyroid hormones in RA was further supported by an immunohistochemical and genetic study conducted by Pörings et al.^18^, which demonstrated that the inflammatory synovial environment in RA could reduce TH bioavailability by degrading them in situ via deiodinases.

Li et al.^2^, in a case–control study and a meta-analysis, reported a significantly higher frequency of both hypothyroidism and hyperthyroidism in patients with RA compared to healthy controls. These findings are further supported by Liu et al.^15^ in another recent meta-analysis.

A meta-analysis conducted by Pan et al.^14^, which included 13 studies, suggested that thyroid autoimmunity is significantly more common in patients with RA than in healthy controls. In the literature, the frequency of TAAs positivity in RA ranges from 4 to 32%^19^, which is consistent with our study, where 9 patients (representing 15.5%) were positive for TAAs. The prevalence of TPOAb ranges between 5 and 37%^20,21^; in our study, 6 patients (10.3%) tested positive for TPOAb, aligning with the lower range of reported values. Similarly, the prevalence of TgAb between 5 and 31%^21–23^, with our study finding 3 patients (5.2%) positive, which is also at the lower end of this range. Few studies have measured TRAb, with positivity rates ranging from 0 to 7% in RA patients^6,22^; in our study, 2 patients (3.4%) were positive, consistent with the lower spectrum of reported frequencies. Numerous studies have shown that the prevalence of hypothyroidism (primarily subclinical hypothyroidism) and thyroid autoimmunity increases with age in RA patients^24–26^. In our study, we didn’t find a significant difference in age between patients with thyroid dysfunction and those with normal thyroid function. This finding is consistent with that of Tekaya et al.^27^ and Atzeni et al.^9^.

Other than the age of the patients, a link was reported between the occurrence of an autoimmune thyroid disease and the evolution of the rheumatic disease. In fact, Waldenlind et al.^28^ observed that the risk of autoimmune thyroid disease increased during the five years preceding RA diagnosis, peaked at the time of diagnosis, and then declined two to five years after the RA diagnosis. This suggests a potential impact of antirheumatic therapies on reducing the risk of thyroid dysfunction. In our study, neither the age of onset nor the duration of RA was associated with thyroid dysfunction or the presence of TAAs. Similar findings were reported by Li et al.^2^, Emamifar et al.^29^, and Atzeni et al.^9^, who found no correlation between disease duration and thyroid status in RA.

Studies on the relationship between RA disease activity and thyroid status have reported conflicting results. In our study, no statistically significant association was found between thyroid status and the DAS28 score, nor with the Visual Analog Scale (VAS), swollen and tender joint counts, radiographic scores, or rheumatoid nodules. Similarly, no significant association was found between thyroid status and the functional impact of the disease. There was no correlation between thyroid autoimmunity and disease activity or functional capacity. Like our results, other studies have not demonstrated an association between thyroid status and RA disease activity, including those by Tekaya et al.^27^, Atzeni et al.^9^, and Hussein et al.^30^. In contrast, Waseem et al.^31^ reported a significant association between thyroid dysfunction and RA disease activity. Disease activity parameters such as VAS for pain, the Global Assessment Scale (GAS), swollen joint counts, DAS28, and erythrocyte sedimentation rate (ESR) were significantly higher in patients with thyroid dysfunction compared to those without. In the study by Waldenlind et al.^5^, HAQ and pain levels were significantly higher in patients with clinical autoimmune thyroiditis compared to those without at the time of RA diagnosis. The authors suggested that in RA, patient-reported outcomes like VAS and HAQ might be overestimated due to symptoms like fatigue, arthralgia, and myalgia caused by concurrent thyroid disorders. Posselt et al.^32^ found a significant association between TPOAb, hypothyroidism, and RA disease activity.

In our study, 87% of patients were RF positive, and 73% were positive for ACPA. However, no significant association was found between thyroid status and the serological status of patients. Comparable results have been reported in several studies, such as those by Joshi et al.^33^, Cardenas Roldan et al.^20^, and Koszarny et al.^6^. Nadeem et al.^34^ also conducted a cross-sectional study on 385 RA patients, reporting a higher frequency of RF positivity in subjects with subclinical hypothyroidism compared to those without. While no significant association was found between ACPA and the thyroid status^34^. Different results were found in the study by Ghitany et al.^35^, who compared the thyroid function in RA patients based on seropositivity (RF and/or ACPA) versus Sero negativity. Although no difference was found between the two groups in terms of thyroid dysfunction frequency, a higher prevalence of thyroid autoimmunity was observed in seropositive RA patients compared to seronegative ones. Indeed, TPOAb and TRAb levels were positively correlated with RF and ACPA levels. Similarly, Karakiliç et al.^36^ found a positive correlation between TPOAb and ACPA levels. These findings support the hypothesis that RA and thyroid dysfunction share a common autoimmune process.

In our study, no association was found between thyroid status and CRP levels in RA patients. These findings are consistent with those of El Attar et al.^37^ and Atzeni et al.^9^. However, Koszarny et al.^6^, in their study on the relationship between RA activity and thyroid autoimmunity, found a positive correlation between plasma TgAb levels, CRP, and ESR. A positive and significant correlation between serum TSH levels and ESR was reported by Azeem et al.^38^ in a study exploring the impact of hypothyroidism on 1000 RA patients. However, no correlation was found between thyroid hormones and CRP levels. These findings align with those reported by Saqre et al.^39^, where RA patients with comorbid autoimmune thyroid disease had significantly higher ESR levels than those without thyroid dysfunction, but no difference in CRP levels was found between the two groups.

The strengths of our study include being the first in Tunisia to measure anti-TSH receptor antibodies in RA patients, as well as performing tests for thyroid autoantibodies that are not routinely measured in clinical biochemistry laboratories. However, the small sample size may limit the statistical power of our findings. Genetic predisposition to thyroid disorders was not assessed in this study, which represents another limitation.

In conclusion, thyroid disorders are common in the general population, but they are even more prevalent in patients with autoimmune diseases like RA. The elevated risk of thyroid dysfunction in RA highlights the importance and relevance of conducting thyroid evaluations. However, the thyroid dysfunction and the presence of antithyroid antibodies did not influence the course of the RA. Larger studies should be done before concluding.

## Data Availability

The datasets generated and analyzed during the current study are not publicly available due to restrictions on ethical and data management approvals. Still, they are available from the corresponding author upon reasonable request.
